# Impact of Different Type and Frequency of Social Participation on Depressive Symptoms Among Older Chinese Adults: Is There a Gender Difference?

**DOI:** 10.3389/fpsyt.2021.758105

**Published:** 2021-09-30

**Authors:** Shujuan Xiao, Huang Lin, Chongbang Zhao, Xiao Zheng, Lei Shi, Jiachi Zhang, Benli Xue, Jinghui Chang, Jiangyun Chen, Chichen Zhang

**Affiliations:** ^1^School of Health Management, Southern Medical University, Guangzhou, China; ^2^Department of Psychiatry, The Third Affiliated Hospital, Sun Yat-sen University, Guangzhou, China; ^3^Department of Health Management, Nanfang Hospital, Southern Medical University, Guangzhou, China; ^4^Institute of Health Management, Southern Medical University, Guangzhou, China

**Keywords:** depression, social participation, gender differences, older adults, Chinese

## Abstract

**Objectives:** Social participation may prevent depressive symptoms in older adults. But research to date ignores gender differences in the associations between social participation and depressive symptoms. The purpose of this study was to determine the effect of different type and frequency of social participation on depressive symptoms, as well as if there is a gender difference in these correlations among older Chinese adults.

**Methods:** Data was obtained from adults aged 60 years or above in the 2018 China Health and Retirement Longitudinal Survey, a nationally representative sample of older adults in China. Depressive symptoms were measured using CESD-10. Social participation included participation in social groups, hobby groups, sports groups, and community-related organizations. The independent relationships between each type of social participation and depressive symptoms were assessed using multiple linear regression models.

**Results:** A total of 6,287 older adults were included in this analysis, of whom 49.69% were women. Participating in social groups, sports groups, and community-related organizations with a frequency of one or more per week was all linked to better mental health. Furthermore, our findings suggest that the positive relationship between participation in social groups, hobby groups, and community-related organizations and depressive symptoms is more flexible for older men than for women.

**Conclusions:** Older individuals who participate in social participation at a high frequency may have better mental health. The findings provide novel insights into mental health from the standpoint of social participation in older adults. Gender differences in the associations between social participation and depressive symptoms need to be considered when formulating interventions to prevent depression.

## Introduction

Depression, one of the most frequent psychiatric illnesses, affects around 7% of the world's older population (1). According to a meta-analysis, the incidence of depressive symptoms among older Chinese adults aged 60 or above is as high as 22.7 %, with more than 90% not receiving quick and regular treatment (2). Depressive symptoms not only reduce one's quality of life, but it also has a financial impact on society and families (3, 4). Depression is more likely to be associated with suicide in older individuals than in any other age group (5). Depression was predicted by the World Health Organization to become the second largest cause of mortality in older adults, endangering their health and safety (6). Given the deteriorating course and consequences of depression, it is critical to discover effective methods of preventing and treating depression in older adults.

The social participation of older adults is receiving increasing attention as one of the three pillars of active aging. Social participation has grown in importance as a means of gaining social resources (7). Taking on responsibilities gives older people a feeling of meaning and purpose, which improves their mental health. Depressive symptoms are reduced by active social participation in old age (8, 9). In recent years, a growing number of studies have discovered that social participation has piqued the interest of researchers due to its low cost and widespread availability (10, 11). It was noted that the influence of social participation on the health of older people varies greatly depending on the type of group involved (12). However, little is known about which type and at what frequency of social participation impact the risk of depressive symptoms among older adults. These findings encouraged us to investigate the impact of various types and frequency of social participation on depression in Chinese older adults.

Previous studies have discovered that older males and females had different levels of mental health and social participation. Gender differences in mental health are largely constant across studies, with older females suffering from greater depression than older male adults (13, 14). Prior research revealed the disparities in depression prevalence between men and women (15). Previous research on social participation has discovered a gender difference in several forms of social participation (16). Furthermore, greater levels of social participation may benefit elderly women more than older men (17). As a result, we hypothesize that older male and female individuals may have different degrees of social participation and depression, as well as different effects of social participation on depression.

Given the importance of social participation in mental health, this study investigated the link between different type and frequency of social participation and depressive symptoms in people aged 60 and older, as well as whether there is a gender difference in these correlations. From a theoretical perspective, we propose the following hypotheses: Hypothesis 1: Social participation has a positive effect on the mental health of older adults. Hypothesis 2: Different social participation types and frequency have different effects on the mental health of older adults. Hypothesis 3: There are different mental health effects on older adults per gender based on social participation. The findings will aid in the development of programs to promote active and healthy aging in older adults.

## Methods

### Study Design and Participants

China Health and Retirement Longitudinal Study (CHARLS) national survey of wave four was conducted from August, 2018 to March, 2019. The CHARLS is a high-quality, nationally representative longitudinal survey of Chinese people aged 45 years or older. Face-to-face interviews were conducted to collect information. The multi-stage probability-proportional-to-size methodology was used to select participants in four stages randomly (18). In the first stage of sampling, 150 counties were randomly selected as representative socioeconomic and geographic areas in China. Then, three main sampling units were selected according to their population. Following that, all households were mapped in each selected main sampling unit, and 24 households were randomly selected from all households as samples. Finally, participants eligible for the age requirement were selected. The inclusion criteria for this study were as follows: (1) being aged 45 and older, with no upper age limit, and (2) having barrier-free communication skills. Those who had difficulty communicating were excluded.

The fourth national survey included 19,816 participants. We applied to the online CHARLS database in December 2020, and it was approved quickly. We limited our sample to participants aged 60 years or older. For the handling of missing data, the simple deletion method was used to delete the cases with missing variables. The final sample contained 6,287 individuals, with 3,163 older male adults and 3,124 older female adults.

### Symptoms of Depressive Symptoms

The Center for Epidemiological Studies Depression Scale-10 (CESD-10) was used in the CHARLS questionnaire to assess depressive symptoms (18). To ensure that respondents understood the content of the scale, enumerators were trained on how to correctly interpret the scale entries in the local language so that respondents could accurately understand the scale entries and enumerators correctly recorded the interviews. The CESD-10 consisted of 10 questions about depressive symptoms, with four possible answers: (1) rarely, (2) some days (1–2 days per week), (3) occasionally (3–4 days per week), and (4) most of the time (5–7 days per week). Total CESD-10 scores ranged from 0 to 30, with higher scores indicating higher depressive symptoms. Previous studies have suggested that the cut-off point for depressive symptoms among older adults is 10 for the CESD-10 scale (19). Cronbach's α for the total scale was 0.786, indicating good reliability and consistency. The CESD-10 has highly validated reliability and validity for older adults in China (20, 21).

### Social Participation

Social participation is the primary explanatory variable in this research. The CHARLS questionnaire inquired about individuals' engagement in 10 social activities throughout the previous month. There were 12 possible answers to this question: (1) interacted with friends, (2) played mahjong, chess, or cards, or attended a community club, (3) assisted family, friends, or neighbors who did not live with them, (4) attended sports, social, or other kinds of clubs, (5) participated in a community-related organization, (6) did voluntary or charity work, (7) cared for a sick or impaired adult who did not live with them, (8) attended an educational or training course, (9) invested in stocks, (10) utilized the Internet, (11) other, or (12) none of these in the last month. Based on the mainstream social participation classification (7, 22), in this study, we examined four types of formal social participation: (1) social groups: interacted with friends, (2) hobby groups: played mahjong, chess, or cards or went to a community club; (3) sports groups: went to sports, social, or other kinds of clubs; and (4) community-related organizations: took part in a community-related organization.

The frequency of social participation was graded as never, not regularly, almost every week, and almost daily. According a prior study (23), we categorized the answers of “almost daily” and “almost every week” into one group as “once or more each week.” Therefore, the frequency measure of each social participation in this study was coded at three levels: 0 = none, 1 = occasionally, and 2 = one or more each week.

### Control Variables

Based on previous research, we described the possible confounders of social participation and depression (24–27). Gender, age, marital status, residence, educational level, sleep duration, chronic pain, chronic diseases, and physical disabilities were all possible confounders. These were chosen because prior research has linked them to social participation and depression. Self-reported sleep duration was obtained via a structured questionnaire with the question “During the past month, how many hours of actual sleep did you get at night (average hours for one night)?”. Self-reported data on chronic diseases was supplemented with diagnostic evidence from medical records or physician prescriptions. Chronic conditions included hypertension, dyslipidemia, diabetes, cancer, chronic lung disease, liver disease, heart disease, stroke, kidney disease, stomach disease, psychiatric problems, memory related disease, arthritis, and asthma due to the design of the survey. Chronic pain was assessed by asking participants whether they were troubled by any such body pains. Chronic pain was recorded as positive if participants responded “yes,” and negative if they responded “no.” Physical disability status was assessed with the question “Do you have one of the following [physical disabilities, brain damage/mental retardation, vision problem, hearing problem, speech impediment] disabilities?” Responses were recoded as “yes” or “no.”

### Data Analysis

All analyses were conducted using Stata 16 (StataCorp, College Station, TX, USA). First, all study variables were analyzed using descriptive analysis. To assess the normality of continuous variables, the Kolmogorov-Smirnov test was utilized. Characteristic differences were examined using the Student's *t*-test for continuous variables and the chi-squared test for categorical variables. The coefficients of social participation were then examined using multiple linear regression models, and separate correlations between each category of social participation and depressive symptoms were estimated. We introduced the multiplicative interaction term (social participation × gender) to assess the heterogeneity of gender across the four categories of social participation and depressive symptoms. The models were adjusted for gender, age, marital status, residence, educational level, physical activity, chronic pain, and chronic diseases. The test level was two-sided, with a *P* < 0.05 deemed statistically significant.

## Results

### The Distribution of CESD-10 Scores by Gender

**Figure 1:**
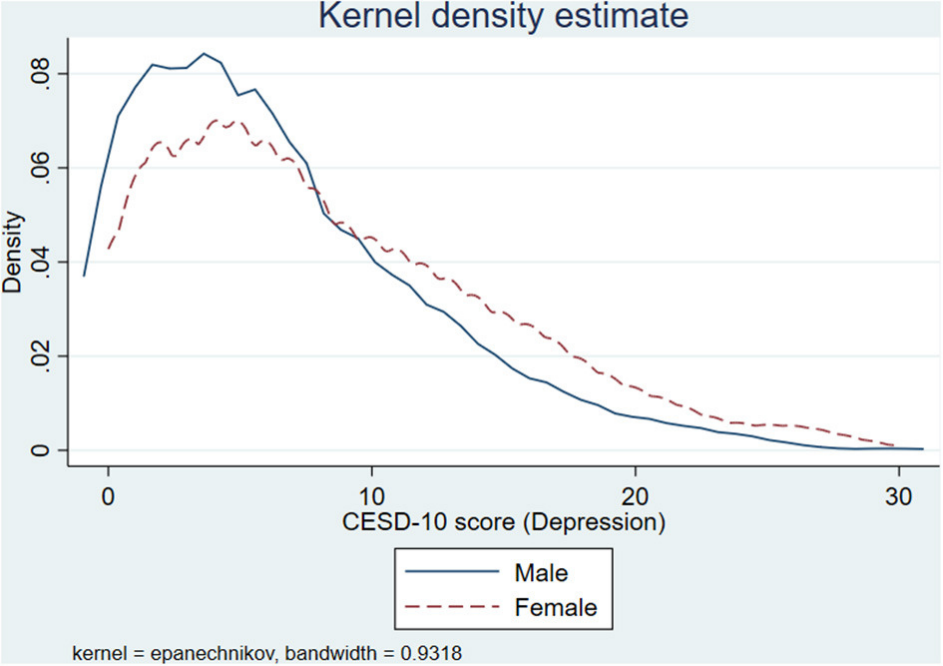
The distribution of CESD-10 scores by gender.

A total CESD-10 score of ≥10 was used to identify patients with depressive symptoms (19). The distribution of CESD-10 scores in older male and female individuals was depicted in Figure 1. Using a score of ~10 as a cut-off point, the right side of the score for older women was higher than that of male adults, implying that older female adults had poorer mental health than male ones.

### Participants' Characteristics

Table 1 presents the characteristics of older adults according to gender. Of the 6,287 participants, 49.69% were women. The respondents' average age was 71.83 years, and the majority of them were married. In terms of socioeconomic status, male participants tended to have a greater degree of educational when compared to female participants. Most of the participants were from rural areas. With respect to the life behaviors of participants, female individuals were more likely to have physical activity than male ones. Furthermore, female participants experienced a greater proportion of chronic pain than male participants. More females in the study had divorced/unmarried/widowed status than males with a significant *P*-value.

**Table 1:** Participants' characteristics.

**Variables**	**All**	**Male, *n* (%)**	**Female, *n* (%)**	***P*-Value**
Total	6,287	3,163 (50.31)	3,124 (49.69)	
CESD-10 score, mean (SD)	7.46 (6.03)	6.59 (5.48)	8.34 (6.42)	<0.001
**Demographic variables**				
*Age (y)*	71.83 (6.55)	71.97 (6.50)	71.69 (6.60)	0.085
*Marital status*				<0.001
Divorced/widowed/Unmarried	721 (11.47)	300 (9.48)	421 (13.48)	
Married	5,566 (88.53)	2,863 (90.52)	2,703 (86.52)	
**Socioeconomic status**				
*Residence*				<0.001
Urban	1,408 (22.40)	650 (20.55)	758 (24.26)	
Rural	4,879 (77.60)	2,513 (79.45)	2,366 (75.74)	
*Educational level*				<0.001
Illiteracy	3,759 (59.79)	1,614 (51.03)	2,145 (68.66)	
Primary school	2,357 (37.49)	1,436 (45.40)	921 (29.48)	
High school or above	171 (2.72)	113 (3.57)	58 (1.86)	
**Life behavior habit**				
Nighttime sleep hours, mean (SD)	6.50 (1.76)	6.59 (1.70)	6.41 (1.81)	<0.001
**Health status**				
*Chronic pain*				<0.001
No	3,263 (51.90)	1,865 (58.96)	1,398 (44.75)	
Yes	3,024 (48.10)	1,298 (41.04)	1,726 (55.25)	
*Chronic diseases*				0.660
No chronic condition	3,680 (58.54)	1,838 (58.11)	1,842 (58.96)	
One chronic disease	1,577 (25.08)	809 (25.58)	768 (24.58)	
Two or more chronic diseases	1,030 (16.38)	516 (16.31)	514 (16.46)	
Physical disabilities				<0.001
No	6,049 (96.21)	3,009 (95.13)	3,040 (97.31)	
Yes	238 (3.79)	154 (4.87)	84 (2.69)	
**Social participation**				
*Social groups*				0.813
None	3,988 (63.43)	2,017 (63.77)	1,971 (63.09)	
Occasionally	733 (11.66)	369 (11.67)	364 (11.65)	
One or more each week	1,566 (24.91)	777 (24.56)	789 (25.26)	
*Hobby groups*				<0.001
None	5,156 (82.01)	2,489 (78.69)	2,667 (85.38)	
Occasionally	300 (4.77)	184 (5.82)	116 (3.71)	
One or more each week	831 (13.22)	490 (15.69)	341 (10.91)	
*Sports groups*				<0.001
None	5,706 (90.76)	2,982 (94.28)	2,724 (87.20)	
Occasionally	69 (1.10)	19 (0.60)	50 (1.60)	
One or more each week	512 (8.14)	162 (5.12)	350 (11.20)	
*Community-related organization*				<0.001
None	5,981 (95.14)	2,956 (93.46)	3,025 (96.83)	
Occasionally	82 (1.30)	52 (1.64)	30 (0.96)	
One or more each week	224 (3.56)	155 (4.90)	69 (2.21)	

Regarding social participation, the high level of participation of older adults in social groups was attributed to the lower physical and residential requirements for this type of social participation. It also reflects the strong demand for emotional and social activities among the elderly. Generally, most participants have never participated in sports or community-related organizations. According to the findings, older male individuals were more likely to be active in hobby groups and community-related organizations, whereas older females were more likely to be involved in social groups and sports clubs.

Regarding gender differences in social participation rates, we can see from Table 1 that the participation rates of social groups were equal for older male and female adults. The participation rate of hobby groups and community-related organizations was higher for older males than for older female adults. However, female seniors had a higher participation rate in sports groups than male seniors.

### Social Participation and Depressive Symptoms

Table 2 presents the multiple regression results for the CESD-10 scores. In Model 1, we find that, with the exception of social groups, sport groups, and community-related organizations with the frequency of occasionally, all four categories of social participation variables are substantially adversely linked with CESD-10 scores (supporting Hypothesis 1). However, the sign is positive for social groups, sport groups, and community-related organizations with the frequency of occasionally, but this relationship was not statistically significant. Model 2 includes the participants' demographic information. When the estimates in Model 2 are compared to the estimates in Model 1, the coefficient of community-related organizations with the frequency of occasionally becomes significant, and the coefficients of hobby groups and community-related organizations with the frequency of one or more each week fall somewhat. After including socioeconomic status into Model 3, the coefficients of social groups, hobby groups, sports groups, and community-related organizations with the frequency of one or more each week decline but remain significant. Model 4 adds life behavior habit, such as sleep duration, and we find that all the coefficients of social participation groups are lower than those of Model 3. Model 5 is further adjusted for health status, and the coefficients of social groups, hobby groups, sport groups, and community-related organizations with the frequency of one or more each week are weaker in completely adjusted models (Model 5) than in Model 1 and continue to be significant. Female older adults were more likely to suffer from depressive symptoms. Older adults in rural areas were more likely to suffer from depressive symptoms. Those older adults with primary and high school or above education level and below had lower odds of depressive symptoms compared with those who were illiterate. In addition, longer sleep duration was identified as a significant protective factor against depressive symptoms. Regarding the health status, chronic pain, and chronic diseases were shown to be highly associated with depressive symptoms in the full model. As for physical function, those who had physical disabilities had higher odds of depressive symptoms.

**Table 2:** Associations between social participation and CESD-10 scores in the older people: multiple regression results (*n* = 6,287).

**Variables**	**Model 1**	**Model 2**	**Model 3**	**Model 4**	**Model 5**
Social groups (occasionally)	0.118	0.164	0.329	0.323	0.240
Social groups (one or more each week)	−1.045^***^	−1.096^***^	−0.975^***^	−0.853^***^	−0.875^***^
Hobby groups (occasionally)	1.206^***^	1.525^***^	1.638^***^	1.555^***^	1.264^***^
Hobby groups (one or more each week)	−1.030^**^	−0.818^***^	−0.764^***^	−0.711^***^	−0.641^***^
Sports groups (occasionally)	0.146	−0.279	−0.123	−0.024	−0.321
Sports groups (one or more each week)	−1.946^***^	−2.381^***^	−2.038^***^	−1.923^***^	−1.672^***^
Community-related organization (occasionally)	1.138	1.432^**^	1.723^***^	1.485^**^	1.339^**^
Community-related Organization (one or more each week)	−2.879^***^	−2.490^***^	−2.276^***^	−2.202^***^	−2.267^***^
Age		−0.006	−0.001	0.004	0.008
Female		1.677^***^	1.429^***^	1.313^***^	1.068^***^
Married		1.809^***^	1.641^***^	1.544^***^	1.397^***^
Rural			0.483^***^	0.607^***^	0.592^**^
Primary school			−1.456^***^	−1.440^***^	−1.289^***^
High school or above			−1.798^***^	−1.727^***^	−1.664^***^
Sleep duration				−0.698^***^	−0.580 ^***^
Chronic pain (yes)					2.165^***^
One chronic disease					0.653^***^
Two or more chronic diseases					1.986^***^
Physical disabilities (yes)					1.078^***^

*** *P < 0.01*;

** P < 0.05; and *P < 0.1.

Specifically, older individuals who engaged social groups one or more each week had CESD-10 scores that were 0.875 points lower than non-participants. Participants in hobby groups one or more each week received an average of 0.641 points less on the CESD-10 than non-participants. Participants in sports groups one or more each week received an average of 1.672 points less on the CESD-10 than non-participants. Individuals who engaged in community-related organizations one or more each week scored 2.267 points lower on the CESD-10 than those who did not (supporting Hypothesis 2).

### The Interaction Effect Between Gender and Social Participation on Depressive Symptoms

**Figure 2:**
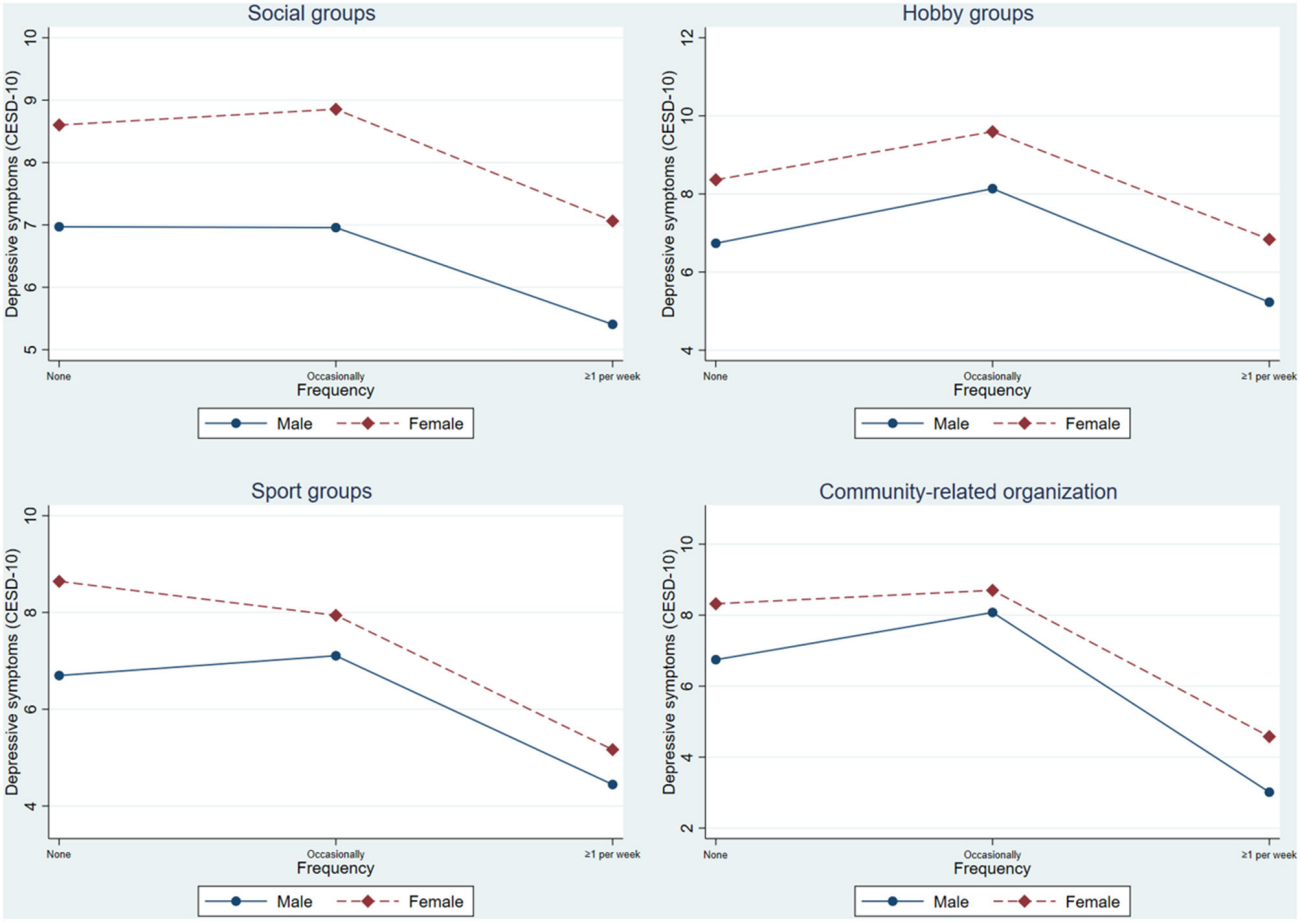
The interaction effect between gender and social participation on CESD-10 scores among older adults.

To examine the heterogeneity of gender, interaction variables between gender and social participation are included in Table 3. This interaction is also depicted in Figure 2, and the significant interaction between gender and social participation was deconstructed by calculating the basic slopes of gender on occasionally and one or more each week frequencies of several categories of social participation. Older female individuals who participate in social groups are significant in both the occasionally and one or more each week frequency categories in Model 6. The findings were positive, suggesting that the link between social groups and depressive symptoms was stronger in males than in females. In Model 7, the effect of hobby groups is significantly stronger among older male adults. The coefficient of the interaction between sports groups and gender becomes insignificant in Model 8. Those older female adults who engaged in community-related organizations were substantially more likely to be in the frequency category of one or more each week in Model 9, and the sign was positive (supporting Hypothesis 3).

**Table 3:** The interaction effect between gender and social participation on CESD-10 scores in older people (*n* = 6,287).

**Variables**	**Model 6**	**Model 7**	**Model 8**	**Model 9**
Social groups (occasionally)	0.018	0.239	0.236	0.236
Social groups (one or more each week)	−0.852^***^	−0.872^***^	−0.875^***^	−0.877^***^
Hobby groups (occasionally)	1.267^***^	1.101^***^	1.285^***^	1.263^***^
Hobby groups (one or more each week)	−0.647^***^	−0.647^**^	−0.652^***^	−0.634^***^
Sports groups (occasionally)	−0.347	−0.320	−0.404	−0.351
Sports groups (one or more each week)	−1.671^***^	−1.677^***^	−0.866^*^	−1.712^***^
Community-related organization (occasionally)	1.324^**^	1.339^**^	1.374^**^	1.084
Community-related Organization (one or more each week)	−2.273^***^	−2.267^***^	−2.334^***^	−2.614^***^
Social groups (occasionally)*female	1.473^***^			
Social groups (one or more each week)*female	0.984^***^			
Hobby groups (occasionally)*female		1.462^**^		
Hobby groups (one or more each week)*female		1.058^***^		
Sports groups (occasionally)*female			1.236	
Sports groups (one or more each week)*female			−0.039	
Community-related organization (occasionally)*female				1.733
Community-related Organization (one or more each week)*female				2.165^***^

*** *P < 0.01*;

** *P < 0.05; and*

* *P < 0.1*.

*All models controlled for age, marital status, residence, educational attainment, sleep duration, chronic pain, chronic diseases, and physical disabilities*.

## Discussion

This study used the CHARLS data in 2018 to explore the effect of social participation on depressive symptoms among older adults. In the current investigation, we discovered that the average CESD-10 score was 7.46 ± 6.03, which was slightly lower than the prior study in China (28). Furthermore, we obtained evidence indicating that older females are more likely to suffer from depressive symptoms, which is consistent with the findings of earlier research (13, 14). The findings mean that public health initiatives to enhance mental health should be gender sensitive, with a special emphasis on older female individuals.

We explored the links between different type and frequency of social participation and depressive symptoms. We found that when we just adjust for demographic factors, the correlations change, indicating that gender or married status may have a moderating impact. Chronic pain, chronic diseases, and physical disabilities were shown to be highly associated with depressive symptoms in the full model. Our results are in accordance with prior research that found that health status influenced by physical conditions can be the main and direct reason for depression (29–31). Our major findings demonstrate that there are substantial connections between the four categories of social participation and depressive symptoms: participating in social groups, hobby groups, community-related organizations, and sports groups with the frequency of one or more each week are connected with a reduced risk of depressive symptoms. Many prior studies have proven the relevance of social participation in older people's health (32, 33).

Scholars have pointed out that participating in social groups, hobby groups, and community-related organizations, and sport groups effectively improves mental health in older people (34). Participating in social groups can assist individuals in gaining greater social support, which may protect against depression (35). Social groups are the most accessible form of socialization for older individuals, particularly in China's humane society. Older individuals who participate in sports and community-related organizations are more likely to be content with their lives and retain good attitudes, resulting in better mental health (36). High levels of participation in sports and community-related organizations have been linked to improved health outcomes, particularly mental health, among older individuals (37). Older adults who participate in sports groups have personal social networks with higher frequencies of interaction and intimacy. Hobby groups are involved in activities such as playing mahjong, chess, cards, and going to community rooms, which are important forms of recreation for the older adults in China. A prior study found that playing mahjong or cards predicted a decline in depressive symptoms in older urban respondents (11). Participating in hobby groups regularly may reduce the risk of depression in older adults by improving social support (38). Participation in community-related organizations can provide older individuals with specific social roles, and the more roles they have, the better their psychological and physical functioning. Thus, we speculate that high frequency of participation in social groups, sports groups, hobby groups, and community-related organizations may reduce the risk of depression among older adults.

We have also found that social groups, hobby groups, and community-related organizations are associated with a higher rate of depressive symptoms of a significantly greater magnitude among older female adults. In contrast, older male adults are comparatively less likely to suffer from depressive symptoms at a high frequency of social participation. One key aspect that may explain the division of gender roles is deeply rooted in ancient Chinese society. According to a Chinese adage, “The man goes out to work while the woman takes care of the house (39).” Traditional Chinese society holds that women should handle more family intramural affairs, while participating in public arena activities is the responsibility of men. After retirement, men are more likely to be “idle” while women are still taking care of household chores. Although China has been promoting gender equality, the division of gender roles has been deeply rooted in the generation of older adults. In addition, most current older adults grew up in a patriarchal era. Combined with the scarcity of higher education resources, most families generally place more emphasis on men's academic education, resulting in a higher degree of education among current male older adults than women. Older adults with higher literacy levels have better financial ability and stronger health awareness, and are also more likely to adopt new health concepts and be open to social participation. As a result, men have better mental health than women. As a result, older male individuals are more likely than older female adults to acquire a feeling of spiritual fulfillment and appropriate social support from social groups, hobby groups, and community-related organizations. It is worth noting that the coefficient of interaction between sports groups and gender became insignificant. As a common form of sport with Chinese characteristics, square dancing is a popular pastime due to its low cost and cheerful atmosphere (40), which also has a significant impact on the mental health of retired older female adults in China.

The sample obtained for this study is representative of the older population in China, and has almost equal male and female representation, so the results of this study are generalizable. However, it must be acknowledged that this study does have several limitations. First, the depression variable in this study was assessed using the CESD-10, with no objective measures. Second, chronic diseases were ascertained by self-report, supported by diagnostic evidence from medical records or physicians' prescriptions, which may lead to unavoidable misclassification. Future studies should link participation in different waves of the CHARLS and investigate the trends and patterns of different types of social participation on depression.

## Conclusions and Implications

In conclusion, this study offered a foundation for developing depression prevention methods and individual preventative measures through social participation, which will most likely provide evidence to enhance mental health among older adults. This study suggests that increasing targeted social participation in older individuals of both genders may successfully enhance mental health in China, which contributes to the hypothesis regarding the relationship between social participation and depression. More research is required to examine the underlying mechanisms behind gender differences in the relationship between social participation and depression.

The findings of this study have several policy implications. First, given that the results of this study emphasized the importance of social participation, the Chinese government should enhance public childcare services and community-based senior care activities to relieve older individuals of the onerous burden of family care. Second, the government should endeavor to create an atmosphere of respect for women in society and enhance the confidence of older female adults in greater social participation with a more positive attitude.

## Data Availability Statement

The original contributions presented in the study are included in the article/supplementary material, further inquiries can be directed to the corresponding author/s.

## Ethics Statement

CHARLS was approved by the Biomedical Ethics Review Committee of Peking University, Beijing, China (IRB00001052–11015). The patients/participants provided their written informed consent to participate in this study.

## Author Contributions

SX conceived the idea. LS and JCha participated in statistical analysis. SX and HL drafted the manuscript. BX, JZ, and JChe edited the paper. CZhan, CZhao, and XZ gave many valuable comments on the draft and polished it. All authors have read and approved the manuscript.

## Funding

This work was supported by the National Natural Science Foundation of China under Grant (No. 71874104), National Key R&D Program of China under Grant (2020YFC2006400), Guangdong Basic and Applied Basic Research Foundation under Grant (No. 2020A1515110369). The sponsor had no role in the design and conduct of the study; collection, management, analysis, and interpretation of the data; preparation, review, or approval of the manuscript; and decision to submit the manuscript for publication.

## Conflict of Interest

The authors declare that the research was conducted in the absence of any commercial or financial relationships that could be construed as a potential conflict of interest.

## Publisher's Note

All claims expressed in this article are solely those of the authors and do not necessarily represent those of their affiliated organizations, or those of the publisher, the editors and the reviewers. Any product that may be evaluated in this article, or claim that may be made by its manufacturer, is not guaranteed or endorsed by the publisher.
